# Nanomaterials for Drug Delivery and Cancer Therapy

**DOI:** 10.3390/nano13010207

**Published:** 2023-01-03

**Authors:** Fiore Pasquale Nicoletta, Francesca Iemma

**Affiliations:** Department of Pharmacy, Health and Nutritional Sciences, University of Calabria, 87036 Rende, Italy

In recent decades, the interest in nanomaterials has grown rapidly for their applications in many research fields, including drug delivery and cancer therapy. Polymer, metal, silica, carbon, and hybrid nanoparticles are different kinds of nanomaterials, which are currently available and utilized in a large number of applications for the treatment of disease. Nanoparticles can often be endowed with suitable functionalities to provide nanoformulations with improved performance, including superior pharmacokinetic profile and treatment efficiency, in tailored applications. 

This Special Issue of *Nanomaterials*, entitled “Nanomaterials for Drug Delivery and Cancer Therapy”, covers the recent advances in the use of nanoparticle systems, with an emphasis on preparation and characterization methods, functionalization chemistry, and their associated applications in drug delivery and cancer therapy. Moreover, some recent advances are presented here, with the aim to provide new ideas and to ignite a discussion among researchers working in this multidisciplinary field.

This Special Issue collects seven recent research articles and four review papers, with the latter being presented at the beginning of the book issue, since they can provide an extensive overview of the topics presented herein, with a discussion of future perspectives.

The first review article, by Jie Tang et al., provides an overview of the recent developments in nucleic acid delivery systems that target airway mucosa for vaccination purposes [1]. The article outlines the appeal of a respiratory mucosal vaccination, but at the same time stresses the challenges of several physical and biological barriers at the airway mucosal site, such as a variety of protective enzymes and mucociliary clearance. The authors discuss in detail the nanotechnologies enabling novel nucleic acid formulations for the efficient delivery of both mRNA transcribed in vitro and nucleic-acid-based vaccines.

In the second review article, Rabia Arshad et al. highlight the latest advances in cancer diagnosis and treatment by RNA nanotechnology, in particular by using multi-functionalized nanoparticles and nanobiosensors with specified ligands able to target the tissues of interest [2]. RNA-conjugated nanomaterials have demonstrated improved sensitivity and selectivity, higher therapeutic efficacy, more accurate diagnosis, lower toxicity, and more site-specific delivery than conventional techniques, resulting in better cytotoxicity management and cost-effectiveness. However, the main drawback of such technology is related to the low number of RNA-based nanoparticles that have progressed to clinical trials.

In the third review article, Shei Li Chung et al. describe the recent advancements in nanotechnology to develop novel co-delivery systems, which involve short-interfering RNA (siRNA) and small-molecule drugs for synergistic cancer therapy [3]. They discuss the problems related to the co-delivery of two distinct anti-tumor agents with different properties, showing some key examples. 

The fourth review paper, by Manuela Curcio and colleagues, reports the possibilities offered by self-assembling nanoparticles based on hyaluronic acid in cancer therapy [4]. Such systems conjugate the targeting activity of hyaluronic acid towards cancer cells with the chemical versatility, ease of preparation and scalability of self-assembling nanoparticles. The authors elucidate the different hyaluronic acid derivatization strategies and preparation methods used for the fabrication of different delivery devices. After showing the biological results in in vivo and in vitro models, the pros and cons of each nanosystem are reported, opening a discussion on which approach could represent the most promising strategy for further investigation and the development of effective therapeutic protocols.

This Special Issue also includes seven research articles.

The first paper, by Nicole F. Bonan et al., presents a novel formulation of Prussian Blue nanoparticles (PBNPs) conjugated with an antibody targeting Fn14, the fibroblast growth factor-inducible 14, which is expressed in glioblastoma cell lines [5]. After having characterized the properties of the conjugate anti-Fn14-PBNPs, the authors report its ability to act as a targeted photothermal therapy agent against glioblastoma. 

The second paper, by Andrew Dunphy et al., provides evidence of the interplay between macrophages and carbon nanodots, which represent a well-known target in therapeutic treatments and an important nanomaterial for biomedical applications, respectively [6]. After having found that CNDs were non-toxic in a variety of doses and that the expression of CD 206 and CD 68 could be altered by opportune treatments, they examined the potential entrance routes of CNDs into macrophages. 

The third paper, by Guan Zhen He and Wen Jen Lin, is focused on the synthesis of a PLGA-PEG-maleimide copolymer, which was used for the encapsulation of Seliciclib [7]. The obtained nanoparticles were decorated with T7 peptide, which is a targeting ligand for the transferrin receptors, generally being overexpressed in cancer cells. The results show that the cellular uptake was, as expected, dependent on the overexpression of transferrin receptors, and that IC50 values were lowered for encapsulated Seliciclib.

The fourth paper, by Brero’s group, aimed to evaluate a novel therapeutic protocol for the in vitro treatment of pancreatic cancer BxPC3 cells [8]. They combined hadron therapy by carbon ions with magnetic fluid hyperthermia and found that the new protocol diminished the clonogenic survival to an extent that depended on the radiation type (using photons as the control), and the decrease was amplified both by the hyperthermia protocol and the cellular uptake of magnetic nanoparticles.

The fifth paper, by Dina Farrakhova and co-workers, presents experimental evidence of a new spectroscopic approach to determine the state of a brain tumor and its microenvironment via changes in the fluorescence lifetime of Indocyanine Green (IG) [9]. In particular, IG accumulated in the tumor site with a maximum accumulation at 24 h after systemic administration and led to different values of fluorescence lifetimes for ICG and ICG aggregates, indicating promising suitability for the fluorescent diagnosis of brain tumors.

In the sixth paper, Mei-Hsiu Chen et al. synthesized a complex, Au–OMV, with gold nanoparticles (AuNPs) and outer-membrane vesicles (OMVs) [10]. Au–OMV, when combined with radiotherapy, produced radiosensitizing and immunomodulatory effects that successfully suppressed tumor growth in both subcutaneous G261 tumor-bearing and in situ (brain) tumor-bearing C57BL/6 mice. Longer survival was also noted for in situ tumor-bearing mice treated with Au–OMV and radiotherapy. The mechanisms behind the successful treatment were evaluated. 

Finally, the seventh paper, by Manuela Curcio’s group, investigated new dual pH/redox-responsive nanoparticles with an affinity for folate receptors, prepared by the combination of two amphiphilic dextran (DEX) derivatives [11]. The first derivative was obtained from a covalent coupling of the polysaccharide with folic acid (FA), whereas the second was derived from the reductive amination step of DEX, followed by condensation with polyethylene glycol 600. After self-assembling, nanoparticles could be destabilized in acidic pH and reducing media, and after doxorubicin loading, the proposed system was able to modulate the drug release in response to different pH and redox conditions. Then, viability and uptake experiments on healthy (MCF-10A) and metastatic cancer (MDA-MB-231) cells proved the potential applicability of the proposed system as a new drug vector in cancer therapy.

We hope that readers enjoy these selected contributions.

## References

[1] Tang J., Cai L., Xu C., Sun S., Liu Y., Rosenecker J., Guan S. (2022). Nanotechnologies in Delivery of DNA and mRNA Vaccines to the Nasal and Pulmonary Mucosa. Nanomaterials.

[2] Arshad R., Fatima I., Sargazi S., Rahdar A., Karamzadeh-Jahromi M., Pandey S., Díez-Pascual A.M., Bilal M. (2021). Novel Perspectives towards RNA-Based Nano-Theranostic Approaches for Cancer Management. Nanomaterials.

[3] Chung S.L., Yee M.S.-L., Hii L.-W., Lim W.-M., Ho M.Y., Khiew P.S., Leong C.-O. (2021). Advances in Nanomaterials Used in Co-Delivery of siRNA and Small Molecule Drugs for Cancer Treatment. Nanomaterials.

[4] Curcio M., Vittorio O., Bell J.L., Iemma F., Nicoletta F.P., Cirillo G. (2022). Hyaluronic Acid within Self-Assembling Nanoparticles: Endless Possibilities for Targeted Cancer Therapy. Nanomaterials.

[5] Bonan N.F., Ledezma D.K., Tovar M.A., Balakrishnan P.B., Fernandes R. (2022). Anti-Fn14-Conjugated Prussian Blue Nanoparticles as a Targeted Photothermal Therapy Agent for Glioblastoma. Nanomaterials.

[6] Dunphy A., Patel K., Belperain S., Pennington A., Chiu N.H.L., Yin Z., Zhu X., Priebe B., Tian S., Wei J. (2021). Modulation of Macrophage Polarization by Carbon Nanodots and Elucidation of Carbon Nanodot Uptake Routes in Macrophages. Nanomaterials.

[7] He G.Z., Lin W.J. (2021). Peptide-Functionalized Nanoparticles-Encapsulated Cyclin-Dependent Kinases Inhibitor Seliciclib in Transferrin Receptor Overexpressed Cancer Cells. Nanomaterials.

[8] Brero F., Albino M., Antoccia A., Arosio P., Avolio M., Berardinelli F., Bettega D., Calzolari P., Ciocca M., Corti M. (2020). Hadron Therapy, Magnetic Nanoparticles and Hyperthermia: A Promising Combined Tool for Pancreatic Cancer Treatment. Nanomaterials.

[9] Farrakhova D., Romanishkin I., Maklygina Y., Bezdetnaya L., Loschenov V. (2021). Analysis of Fluorescence Decay Kinetics of Indocyanine Green Monomers and Aggregates in Brain Tumor Model In Vivo. Nanomaterials.

[10] Chen M.-H., Liu T.-Y., Chen Y.-C., Chen M.-H. (2021). Combining Augmented Radiotherapy and Immunotherapy through a Nano-Gold and Bacterial Outer-Membrane Vesicle Complex for the Treatment of Glioblastoma. Nanomaterials.

[11] Curcio M., Paolì A., Cirillo G., Di Pietro S., Forestiero M., Giordano F., Mauro L., Amantea D., Di Bussolo V., Nicoletta F.P. (2021). Combining Dextran Conjugates with Stimuli-Responsive and Folate-Targeting Activity: A New Class of Multifunctional Nanoparticles for Cancer Therapy. Nanomaterials.

